# Lean on me: attachment and mental health in couples facing cardiovascular disease

**DOI:** 10.3389/fpsyg.2025.1657068

**Published:** 2025-12-04

**Authors:** Karolina Sztajerowski, Paul S. Greenman, Karen Bouchard, Heather Tulloch

**Affiliations:** 1 The Royal Ottawa Hospital, Ottawa, ON, Canada; 2 Division of Cardiac Prevention and Rehabilitation, Prevention and Rehabilitation Centre, University of Ottawa Heart Institute, Ottawa, ON, Canada; 3 Institut du Savoir Montfort, Ottawa, ON, Canada; 4 Department of Psychoeducation and Psychology, Université du Québec en Outaouais, Gatineau, QC, Canada

**Keywords:** romantic attachment, dyadic coping, anxiety, depression, cardiovascular disease

## Abstract

Elevated symptoms of depression and anxiety are common after the onset of cardiovascular disease in both patients and their spouses. Attachment anxiety, attachment avoidance, and the degree to which couples cope jointly with the stress of cardiovascular disease may help to explain why some of them experience worsening psychological distress. The aim of this study was to investigate the link between insecure attachment and the mental health of patients with cardiovascular disease and their spouses, along with the potential mediating role of common dyadic coping (CDC). Patients with cardiovascular disease and their spouses completed validated questionnaires measuring romantic attachment, common dyadic coping, depression, and anxiety. A structural equation modeling framework was used to test an actor-partner interdependence mediation model. Patients’ and spouses’ (*N* = 181 couples; *M* age = 63.15 years; 79% male patients) romantic attachment anxiety was related to their own symptoms of depression and anxiety; the more attachment anxiety they reported, the higher their scores on measures of depression and anxiety were. Patients’ and spouses’ romantic attachment avoidance was related to their own and their spouses’ common dyadic coping, with greater avoidance linked to less common dyadic coping for both. There was no significant relation between common dyadic coping and romantic partners’ mental health. The results suggest that romantic attachment anxiety is related to psychological distress in couples facing cardiovascular disease, and that attachment avoidance is related to low levels of common dyadic coping. Consideration of attachment orientations may be important in the treatment of anxiety and depression among patients and their spouses.

## Introduction

Cardiovascular disease is a hypernym for various types of heart and blood vessel pathologies, including ischemic heart disease, cerebrovascular disease, peripheral vascular disease, and heart failure (Fisher and Scheidt, 2012). Cardiovascular disease is a leading cause of mortality across most Western nations, accounting for 25% of annual deaths in the United States and resulting in $252 billion USD in healthcare expenditures in 2019–2020 (Centers for Disease Control and Prevention, 2024). In Canada, one in 12 adults 20 years of age or older have a diagnosis of heart disease, which represents 2.6 million people (Public Health Agency of Canada, 2024). Ischemic heart disease, which is the weakening of the heart due to decreased blood flow to it, is the most common cardiac affliction in Canada, affecting roughly 8.5% of all adults (Public Health Agency of Canada, 2024). The onset of cardiovascular disease may cause significant functional impairment and psychological distress, including reductions in mobility and symptoms of depression and anxiety. Symptoms such as chest pain and breathlessness can recur or deteriorate, and patients may require secondary preventive interventions like cardiac rehabilitation. In addition to high rates of mortality, rehospitalization, and morbidity, the onset of a cardiovascular condition can prompt social challenges that affect the patient and their family members (Helgeson et al., 2018).

Romantic partners of patients with cardiovascular disease, for example, encounter a plethora of shared stressors, including drastic changes in domestic roles, lifestyle, responsibilities, goals, and life plans (Dalteg et al., 2011; Tulloch et al., 2020). The stress that accompanies the threat of a potentially life-threatening and chronic illness like cardiovascular disease may ultimately endanger the integrity of the couple relationship (Smith and Baucom, 2017; Tulloch and Greenman, 2018). Indeed, approximately 30% of patients with cardiovascular disease report marital discord (Cameron et al., 2017). Reductions in relationship quality in the context of chronic illness has been concurrently and longitudinally linked to poor adherence to treatment regimes, such as pharmaceutical treatments and psychological interventions, and increased symptoms of mental distress (Martire and Helgeson, 2017). On the other hand, supportive relationships may be directly and indirectly (e.g., via reductions in mental health symptoms) linked to reduced cardiovascular disease risk and to more optimal recovery (Smith, 2022; Tulloch and Greenman, 2018).

## Attachment orientations and health outcomes

Attachment theory helps explain why supportive relationships appear to be related to health outcomes, including cardiovascular disease. According to several decades of social psychological research, couple relationships constitute attachment bonds (Zeifman, 2019). Attachment theory posits that human beings are hardwired through evolution to form and to maintain affectional bonds with significant people across the lifespan, including parents during childhood and romantic partners in adulthood, because the establishment and preservation of these emotional ties are crucial to the survival of the human race (Bowlby, 1969; Zeifman, 2019). The assumed function of the attachment system is to promote survival by seeking proximity to these “attachment figures” for comfort in moments of perceived threat or danger (Bowlby, 1973). Thus, “activation” of the attachment system refers to active proximity-seeking with the aim of receiving emotional comfort that will restore a sense of safety and equilibrium.

## Development and activation of the attachment system

According to attachment theory, the quality of early attachment experiences shapes emerging expectations, attitudes, and beliefs about oneself, others, and the larger social world (referred to hereafter as “internal working models;” Bowlby, 1973). A reliable pattern of interactions in which attachment figures behave with responsiveness, availability, and emotional engagement to bids for proximity is known to soothe a person’s distress and deactivate the attachment system, which in turn allows for the pursuit of growth-oriented activities such as exploration and affiliation (Cassidy, 2016). Over time, these repeated experiences of security attainment are thought to be stored at the level of schematic memory and promote the development of a secure attachment bond (Cassidy, 2016). In this way, “securely attached” individuals learn that proximity seeking is an effective emotion regulation strategy that can be confidently and reliably used to relieve distress in times of stress (Simpson and Rholes, 2017).

Conversely, a recurring pattern of interactions in which primary attachment figures lack these characteristics either maintains or heightens a person’s distress and can keep the attachment system activated (Mikulincer and Shaver, 2016). Repeated experiences of security nonfulfillment are also stored at the level of schematic memory, but in this case they foster the development of an “insecure” attachment bond (Mikulincer and Shaver, 2016). Insecurely attached individuals learn that proximity seeking is an ineffective regulation strategy in times of threat or danger and that alternative strategies are needed to relieve distress. These alternative strategies may involve hyperactivation of the attachment system or attempts to deactivate it (Cassidy, 2016). As a result, early attachment experiences provide salient procedural knowledge about distress management, laying the foundation for later intrapersonal development and interpersonal functioning (Mikulincer and Shaver, 2016; Bodenmann, 2008).

## Romantic attachment in adulthood

The internal dynamics of the behavioral attachment system have been found to carry forward into adulthood and to exert a continuing influence in a broad range of social contexts, especially in romantic love (Zeifman and Hazan, 2016). Individuals within a romantic dyad tend to mutually become each other’s central source of support, comfort, and security in times of need (i.e., primary attachment figures), thus making the emotional bond that develops between romantic partners one of the most significant relationships in adulthood. Like the child’s emotional ties to its mother, romantic bonds between adults also appear to have a biological basis and are linked to the survival of the species (Zeifman, 2019).

### Secure vs. insecure attachment in romantic relationships

In this paper, we refer to and measure relationship-specific romantic attachment orientations along the following dimensions: secure, anxious, and avoidant. “Securely attached” romantic partners tend to seek emotional comfort and support from each other in the face of stressors, such as chronic illness (Pietromonaco and Beck, 2019). In contrast, “insecurely attached” romantic partners tend to remain in a state of hyperactivation or deactivation; the person might employ strategies like clinging behaviour in an attempt to regulate their emotions (i.e., “attachment anxiety”) or they may display persistent avoidance of negative affect and demonstrate patterns of withdrawal in their relationships (“attachment avoidance”; Messina et al., 2023).

The two-dimensional conceptualization of adult romantic attachment insecurity is widely recognized as the most comprehensive way of encapsulating the underlying patterns of insecure attachment in adulthood and it reflects the patterns of insecure attachment uncovered in research on children’s relationships (Finkel et al., 2017; Ravitz et al., 2010). The first dimension, “attachment anxiety,” is characterized by negative models of the self that involve excessive fears about partner availability due to persistent self-doubts about one’s worth, lovability, and emotion regulation abilities (Mikulincer and Shaver, 2016). These recurring worries of rejection or abandonment by a romantic partner lead to heightened proximity-seeking efforts, known as “hyperactivating strategies” (Cassidy and Kobak, 1988). In such cases, the distress of anxiously attached individuals becomes amplified as they attempt to obtain attention, protection, and support from their romantic partner and, ultimately, experience a sense of security.

The second dimension of adult romantic attachment insecurity, “attachment avoidance,” is governed by negative models of others which involve fears of emotional dependency and discomfort with interpersonal closeness (Mikulincer and Shaver, 2016). Deeply rooted mistrust about partner availability leads to weakened or blocked proximity-seeking efforts, known as “deactivating strategies.” In such cases, avoidantly attached individuals appear to suppress their distress and engage in compulsive self-reliance to protect themselves against rejection or vulnerability (Simpson and Rholes, 2017). Considering that differences in the organization of the attachment system in adulthood are measured along these dimensions, high scores of either anxiety or avoidance are characteristic of insecure attachment, whereas low scores on both dimensions are indicative of secure attachment (Feeney, 2016). In such cases, securely attached individuals are thought to possess positive models of the self and others, thereby allowing adaptive emotion regulation.

## Romantic attachment and emotion regulation

Health problems like cardiovascular disease tend to activate the attachment system (Simpson and Rholes, 2012) and, depending on their severity and chronicity, the activation of the attachment system may extend over a period of time (Meredith and Strong, 2019). This means that people with chronic illnesses tend to remain in a distressed state, with a salient need for proximity to an attachment figure. This can lead to higher levels of psychological distress, particularly in people with insecure attachment orientations. As previously mentioned, individuals with insecure attachments to their partners are likely to employ hyperactivating or deactivating emotion-regulation strategies in response to stressors, which can affect their responses to physical disease and their mental health.

The results of recent research have indeed borne out the notion of insecure attachment bonds as indicative of ineffective emotion-regulation strategies in the face of stressors. For example, in a recent systematic review of 37 studies of responses to attachment related stressors (e.g., pictures of one’s children with sad faces) with over 2,000 participants, Eilert and Buchheim (2023) found that both attachment anxiety and avoidance were significantly related to difficulties in emotion regulation. People in the studies reviewed who were high in attachment anxiety tended to use fewer cognitive reappraisal strategies, had more impulse regulation problems, and relied more heavily on interpersonal strategies like reassurance-seeking and venting to regulate emotions. Conversely, individuals high in attachment avoidance showed a stronger tendency toward suppression, lower use of reappraisal, and generally less use of interpersonal emotion regulation (Eilert and Buchheim, 2023). Secure attachment, on the other hand, involved more balanced emotion regulation strategies like cognitive reappraisal and effective co-regulation (Eilert and Buchheim, 2023).

Similarly, Messina et al. (2023) found in a recent study of 630 adults in Italy that both anxious attachment to romantic partners was related to impairment in the ability to engage in cognitive appraisal as an emotion-regulation strategy and attachment avoidance was related to attempts to suppress negative emotions rather than to deal with and work though them. Finally, Mosannenzadeh et al. (2024) noted in their study of “inflexible over-reliance on either intrapersonal (self-directed, e.g., suppression) or interpersonal (involving others, e.g., sharing)” (p. 1) emotion regulation, which combined survey and experience-sampling measures, that adults in the Netherlands who demonstrated anxious attachment to their relationship partners tended to turn to their partner for comfort, but they did not adjust that tendency depending on whether their partner was actually available. Even when their partner was not responsive, they still tended to want or to try to seek support, rather than switching to self-soothing strategies. On the other hand, those with avoidant attachment to partners exhibited a tendency to rely excessively on intrapersonal strategies, generally looking solely to themselves for emotional support. All these results demonstrate that ineffective emotion regulation strategies are characteristic of insecure attachment, which can influence the ways in which individuals and their partners cope with the stress generated by chronic illnesses such as cardiac disease.

## Romantic attachment and responses to cardiac illness: links to mental health

There is mounting evidence that insecure attachment to relationship partners and the challenges to potent emotion regulation that it poses can affect how people with cardiac disease and their partners cope with the stress of the illness, which can, in turn have a negative impact on their mental health. This reflects the general finding in the literature that insecure attachment to romantic partners is linked with depression, anxiety, PTSD, and a host of other mental health problems (see Mikulincer and Shaver, 2016, for a review). Meredith and Strong (2019) reviewed the literature on attachment in chronic illness and concluded that the enduring stress and dependency associated with conditions such as diabetes, arthritis, and cardiovascular disease create contexts in which attachment needs become particularly salient, as attachment theory would predict. More recent empirical work has shown that, in cardiac populations, attachment insecurity undermines both patients’ and partners’ adjustment. For example, George-Levi et al. (2020) demonstrated in their study of 114 couples that cardiac patients with higher attachment anxiety showed greater difficulties trusting care, which may have contributed the maintenance of symptoms of anxiety over time following their diagnosis. Avoidant patients in their sample did not appear to seek or respond to partner support (George-Levi et al., 2020). Laflamme et al. (2022) found that couple relationship partners who were providing care for individuals with heart disease reported higher caregiver burden and psychological distress when they were insecurely attached to their partner. In this investigation of 181 patients with heart disease and their life partners, anxiously attached caregivers tended to become overinvolved and distressed by worry, whereas avoidantly attached caregivers appeared to struggle with the sustained intimacy and dependency demands of caregiving, which overwhelmed their usual distancing strategies (Laflamme et al., 2022).

Over time, the continuous activation of the attachment system and difficulties responding effectively to their own and to their partner’s distress may result in an elevated risk for symptoms of depression and anxiety (Mikulincer and Shaver, 2016). To our knowledge, there have been seven studies of the links between attachment orientations and psychological distress in couples facing cardiovascular disease. In four of them (Kidd et al., 2016; Vilchinsky et al., 2010; Heenan et al., 2020; Söllner et al., 2018), attachment was examined as a predictor of psychological distress among patients with cardiovascular disease over the short term (2–3 months), long term (6–12 months), or in response to psychotherapy. The results indicated that attachment anxiety was a strong predictor of depression, anxiety (Heenan et al., 2020; Kidd et al., 2016), and post-traumatic stress (Heenan et al., 2020) in people with cardiovascular disease, and that secure attachment was linked to reductions in depression (Söllner et al., 2018). Attachment avoidance did not appear to be related to mental-health outcomes in these investigations, despite meta-analytic findings in previous studies of significant associations between attachment avoidance and depression (Zhang et al., 2022), anxiety (van Leeuwen et al., 2020), and post-traumatic stress disorder (PTSD) (Ogle et al., 2015).

There are also two additional studies of the role of attachment orientations in psychological distress among spouses of patients with cardiovascular disease. In Laflamme et al.’s (2022) cross-sectional investigation, attachment anxiety and attachment avoidance predicted depression in life partners of patients with cardiovascular disease; this link was mediated by caregiver burden in both cases. In a longitudinal investigation of female partners of male patients with cardiovascular disease, Vilchinsky et al. (2015) determined that attachment-related anxiety moderated the relationship between caregiver burden at one-month post- hospitalization and spouses’ depressive symptoms at six-months follow up. Insights from these studies, however, were gleaned from just one member of the couple, which precludes the possibility of evaluating effects that take place within the dyad.

Interdependent effects of attachment and psychological distress among couples in which one or both partners have cardiovascular disease have been articulated in only one study (Kafescioglu et al., 2010). In it, dyadic coping, defined as the ways in which romantic partners cope together as a unit when dealing with a stressor, was found to be a partial mediator of attachment insecurity and mental health outcomes. Only actor effects were observed in this study. This means that one’s own attachment insecurity and perceptions of dyadic coping were not related to one’s partner’s mental health. The interpretation of these results, however, is limited by a small sample size (*N* = 72 couples), the use of measures with poor psychometric properties, and the exclusion of *common dyadic coping*, which might be a catalyst for the effects of attachment on mental health in people with cardiovascular disease.

In sum, it appears that attachment bonds are important predictors of mental health trajectories. Internalizing symptoms such as depression and anxiety tend to co-occur and are robustly linked to recurrent cardiovascular events and to all-cause mortality among those with established cardiovascular disease (Chen et al., 2019; Meijer et al., 2011). There is also evidence that dyadic coping may be involved in the relation between attachment and mental-health outcomes in patients and partners facing cardiovascular disease (Kafescioglu et al., 2010). It is therefore crucial to identify potentially modifiable factors, including relational ones, that can mitigate the worsening of patients’ and partners’ mental health. Dyadic coping is one such factor.

## Dyadic coping

Cardiovascular disease is a type of “dyadic stress,” which has been defined as any form of stressful life encounter directly affecting the couple as a unit that elicits common concerns and joint appraisals in both romantic partners (Bodenmann, 1997, 2005). Research has found that dyadic stress, from daily hassles to chronic illness such as cardiovascular disease, can affect the mental health and relationship satisfaction of romantic partners, and that effective dyadic coping may buffer against these negative outcomes (Aldwin and Revenson, 1987; Papp and Witt, 2010; Randall and Bodenmann, 2009; Rusu et al., 2016), similar to the way that secure attachment can.

The Systemic Transactional Model (STM) (Bodenmann, 1995, 2005) can be understood as an extension of the Transactional Theory of Stress and Coping (Lazarus and Folkman, 1984) within a romantic perspective. The STM is based upon the postulate that stress has crossover effects from one romantic partner to the other due to the interdependent nature of couple relationships (Bodenmann, 1995). Theoretically, the STM suggests that the resources of a supportive partner may augment the available resources of the stressed partner, thereby reshaping the appraisal of external and/or internal demands as less threatening (Bodenmann, 1995). Dyadic coping is conceptualized in this model as an interpersonal stress regulation process involving both romantic partners dealing jointly with stressors that affect each partner, either directly or indirectly (Bodenmann, 2005).

Emerging research has found that *common dyadic coping* (CDC), which is a specific form of dyadic coping that occurs when both partners conjointly work together towards mitigating or resolving stressors experienced as a dyad by focusing on specific problems (e.g., joint problem-solving) or by engaging in a conjoint effort to regulate emotions (e.g., joint relaxation), is the most salient form of dyadic coping for couples facing stressors (Falconier et al., 2015; Falconier and Kuhn, 2019). Common dyadic coping is used in response to a stressful situation that is initially related to one member of the dyad, such as the onset of cardiovascular disease in an individual (Rapelli et al., 2021), but is experienced as a situation affecting both partners (i.e., a “we-disease”). Other positive forms include *supportive dyadic coping* (i.e., when one partner helps the other to deal with stress, and *delegated dyadic coping* (i.e., when one partner undertakes the responsibilities usually assumed by the other to help relieve the experienced stress) (Bodenmann, 2005). There are also negative forms including *hostile dyadic coping* (i.e., when one partner disparages or criticizes the other), *superficial dyadic coping* (i.e., when one partner offers support in an insincere or inattentive manner), and *ambivalent dyadic coping* (i.e., when one partner offers support reluctantly or grudgingly) (Bodenmann, 2005). There is evidence of links between the various types of dyadic coping and attachment to romantic partners.

## Dyadic coping and adult romantic attachment

Attachment researchers (e.g., Cassidy, 2016; Mikulincer and Shaver, 2016; Simpson and Rholes, 2017) have found that internal working models and strategies for responding to the activation of the attachment system not only shape the ways in which individuals in romantic relationships seek and perceive support from their partner, but they also influence how people in a relationship provide support to their distressed partner. Given that insecure romantic attachment has been found to compound emotion regulation and individual coping difficulties, a growing body of empirical evidence suggests that insecure romantic attachment may also hinder effective dyadic coping (Alves et al., 2019; Batinic and Kamenov, 2017; Gagliardi et al., 2013; Levesque et al., 2017). For instance, Batinic and Kamenov (2017) examined actor and partner effects of insecure romantic attachment on dyadic coping in a sample of 223 opposite-sex, married couples through dyadic analyses. They found significant actor and partner effects (ranging from *β* = −0.11 to *β* = −0.46), indicating that men and women with high levels of attachment anxiety and attachment avoidance were both less prone to engage in CDC themselves and less prone to have a partner who engaged in CDC.

In the same vein, Alves et al. (2019) recently conducted a longitudinal study investigating the mediating effect of CDC between insecure romantic attachment and parental adjustment in a sample of 92 opposite-sex couples. Significant actor and partner effects were found, suggesting that fathers and mothers with high levels of attachment anxiety and attachment avoidance were both less likely to engage in CDC themselves and less likely to have a partner that engaged in CDC. Interestingly, their findings revealed that the associations between attachment anxiety and CDC were broadly weaker in comparison to the associations between attachment avoidance and CDC, suggesting that the excessive worries experienced by individuals with high levels of attachment anxiety may inhibit their ability to engage in joint coping efforts in times of stress.

## Dyadic coping as a mediator between romantic attachment and mental health

In light of theoretical and empirical developments from the past few years, researchers have increasingly started exploring whether dyadic coping may serve as a pathway through which romantic attachment affects intrapersonal and interpersonal outcomes. Although the dyadic coping literature indicates that dyadic coping may be more closely related to relational outcomes than individual outcomes (Bodenmann et al., 2011), dyadic coping has nonetheless been found to mediate the relationship between romantic attachment and intrapersonal outcomes, such as mental health (Kafescioglu et al., 2010), non-suicidal self-injury (Levesque et al., 2017), parental adjustment (Alves et al., 2019), and quality of life (Crangle et al., 2020).

To our knowledge, there has been only one study to date of the ways in which dyadic coping (i.e., supportive and negative dyadic coping exclusively) may be a mediating factor between insecure romantic attachment and mental health. Among a sample of 63 couples in which one partner had received a cardiac diagnosis, Kafescioglu et al. (2010) conducted Actor-Partner Interdependence Model (APIM) analyses through the use of a pooled regression approach. They found two significant mediating effects. On one hand, patients with higher levels of attachment avoidance were more likely to engage in more negative dyadic coping, which in turn appeared to negatively influence their own mental health (*b* = −28.72, *t*(1) = −3.16, *p* < 0.05). On the other hand, spouses with higher levels of attachment anxiety were more likely to engage in more supportive dyadic coping, which in turn, positively affected their own mental health (*b* = −1.85, *t*(1) = −2.45, *p* < 0.05). However, the small sample size in this study, the use of measures with poor psychometric properties, the use of pooled regressions-APIM analyses, and the exclusion of CDC considerably limit our understanding of dyadic coping in a cardiac context.

## The current study

We have established that attachment to romantic partners is related to mental-health outcomes and psychological distress in people with heart disease (e.g., George-Levi et al., 2020; Laflamme et al., 2022; Meredith and Strong, 2019), that secure attachment in couple relationships is likely to coincide with common dyadic coping whereas insecure attachment has been linked to a lesser likelihood of engaging in effective common dyadic coping (e.g., Alves et al., 2019; Batinic and Kamenov, 2017), and that both supportive and negative dyadic coping have been found to mediate the link between insecure romantic attachment and mental health. Since CDC has been found to have predominantly beneficial effects from an individual and relationship perspective and it is the most prominent form of dyadic coping (Falconier et al., 2015; Rusu et al., 2020), we focus in the present paper specifically on CDC as a mediator, using a dyadic approach as a way to account for actor and partner effects.

## Hypotheses

**Figure 1:**
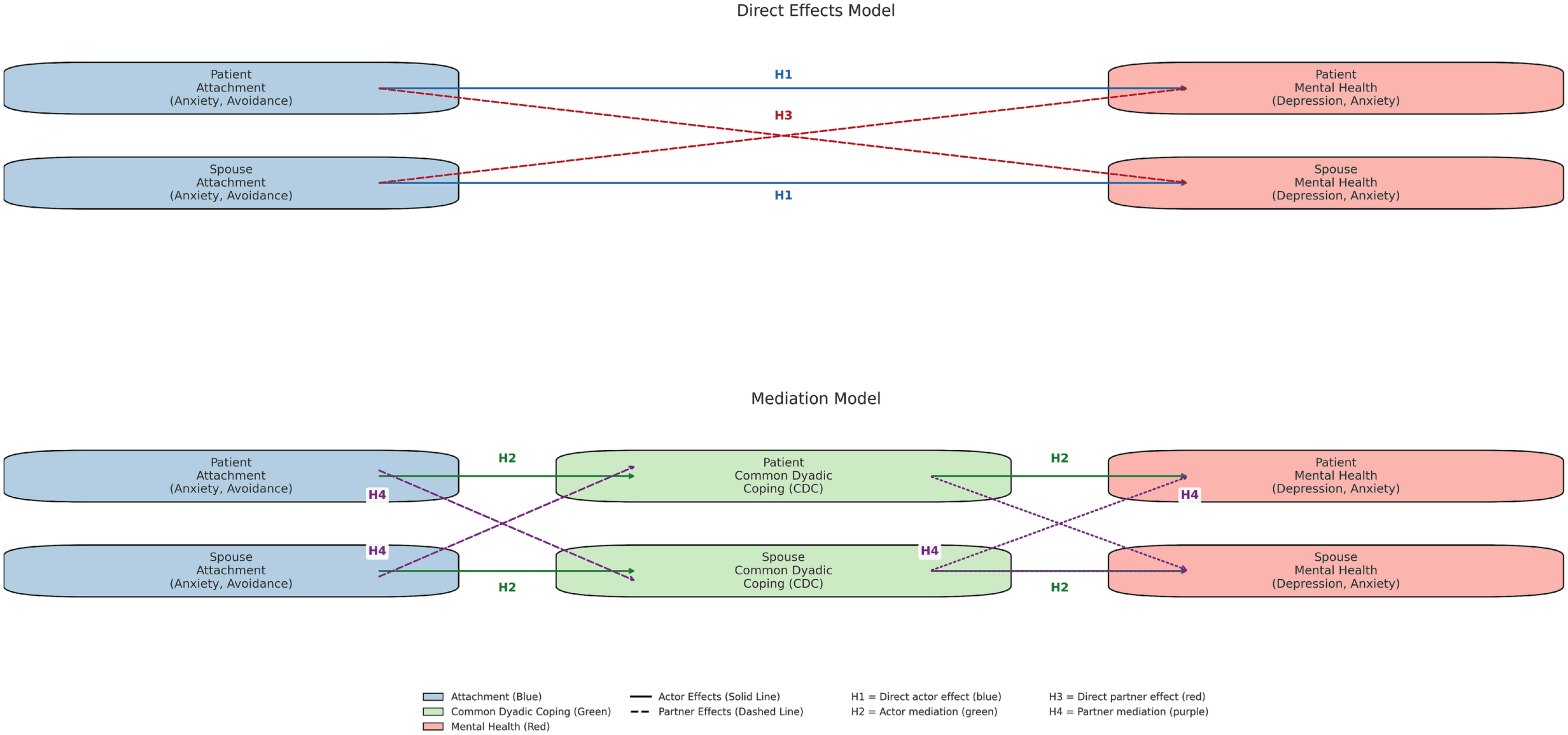
Study hypotheses.

The primary objective of this study was to investigate the link between attachment (attachment avoidance and attachment anxiety) and mental health indicators of anxiety and depression among patients with cardiovascular disease and their spouses. In light of previous findings and the tenets of both attachment theory and the STM, we formulated the following hypotheses:

1) That higher insecure romantic attachment (attachment avoidance and attachment anxiety) would be linked to lower mental health outcomes in both cardiac patients and their partners (*actor effects*);
2) That dyadic coping would mediate the path from attachment security to own mental health. That is, individuals’ own romantic attachment insecurity (attachment avoidance and attachment anxiety) would be linked to their own lower dyadic coping, which in turn would be linked to their own lower mental health (*actor effects*);
3) That individual participants’ own higher insecure romantic attachment (attachment avoidance and attachment anxiety) would be linked to their partners’ lower mental health outcomes (*partner effects*); and
4) That dyadic coping would mediate the path from attachment insecurity to partner mental health. That is, individuals’ higher romantic attachment insecurity (attachment avoidance and attachment anxiety) would be linked to their partner’s lower dyadic coping, which in turn would be linked to their partner’s lower mental health. (*partner effects*). Figure 1 displays all hypothesized pathways.

## Method

### Participants

Participants were recruited (2019–2020) from a cardiac rehabilitation program at a large Canadian academic teaching hospital, serving a population of 1.4 million residents. For context, the program targets patients’ exercise rehabilitation and includes curricula on diet and nutrition. Patients’ mental health is not directly targeted in the cardiac rehabilitation program; patients are referred to outpatient psychological services or a stress management program based on need. Partners are not directly included in the cardiac rehabilitation program but are invited to attend information sessions. Eligibility criteria for the study included: (a) experiencing a cardiac event within the past 6 months, (b) romantic involvement with a partner for at least 1 year, (c) cohabitating with their partner for a minimum of 6 months, (d) being 18 years of age or older, and (e) the ability to read and/or speak English or French.

**Figure 2:**
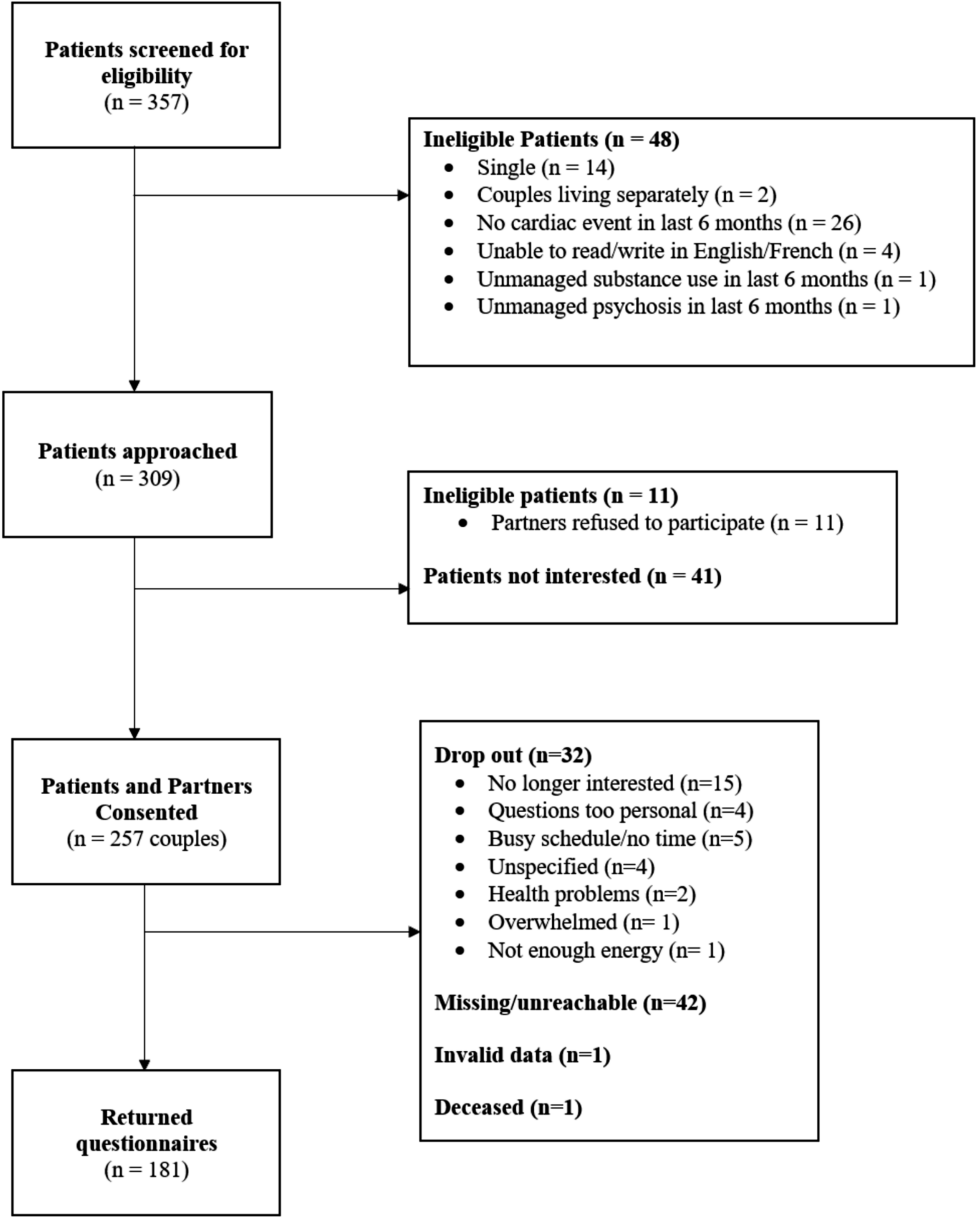
Study flow chart.

A total of 309 eligible couples were approached to participate in the present study. From this sample, 257 patient and spouse dyads consented to participate (83%), and 181 patient and spouse dyads (70% of consented couples) returned completed questionnaires. Thus, the final sample included 181 dyads (see Figure 2 for the study flow chart). The average age of participants was 63.15 years (SD = 10.32). Most patients were male (*n* = 143, 79%), and most of them were diagnosed with coronary artery disease (*n* = 120, 66.3%). Most spouses were women (*n* = 134, 74%). The average length of romantic relationships was 33.70 years (SD = 14.89). The majority of couples were married (*n* = 155, 85.6%) and had children with their current spouse (*n* = 123, 68%). The sample was comprised mostly of heterosexual couples (*n* = 173, 95.6%). Given that the final sample was within the 100–200 range recommended for APIM models by Ledermann et al. (2011), we proceeded with our analyses. Sample characteristics are summarized in Table 1.

**Table 1:** Sample characteristics.

Characteristics	Patients	Couple	Spouses
	M, SD	M, SD	M, SD
Age (years)	64.46 (9.99)		61.76 (10.49)
Sex			
Female	38 (21%)		134 (74%)
Male	143 (79%)		44 (24.3%)
Relationship length (years)		34.03 (14.86)	
Marriage length (years)		33.08 (14.84)	
Cohabitation length (years)		31.27 (14.79)	
Marital status			
Married		155 (85.6%)	
Common law		26 (14.4%)	
Number of children			
With current partner		2.28 (0.82)	
With previous partner	2.3 (0.98)		1.86 (0.87)
Highest education level attained			
University	81 (44.8%)		81 (44.8%)
College	43 (23.8%)		41 (22.7%)
Trade certification	15 (8.3%)		6 (3.3%)
High school	35 (19.3%)		49 (27.1%)
Elementary school	3 (1.7%)		1 (0.6%)
None	3 (1.7%)		1 (0.6%)
Missing	1(0.6%)		2 (1.1%)
Ethnicity			
Caucasian/White	163 (90.1%)		163 (90.1%)
African/Black	2 (1.1%)		3 (1.7%)
Latino/Hispanic	2 (1.1%)		2 (1.1%)
Asian	6 (3.3%)		7 (3.9%)
Middle Eastern	1 (0.6%)		1 (0.6%)
Aboriginal	1 (0.6%)		1 (0.6%)
Other	3 (1.7%)		2 (1.1%)
Primary cardiac diagnosis			
CAD	120 (66.3%)		-
Arrythmia	15 (8.3%)		-
CHF	21 (11.6%)		-
Valve	20 (11%)		-
Congenital HD	2 (1.1%)		-
Aortic aneurysm	3 (1.7%)		=

CAD, Coronary Artery Disease; CHF, Congestive Heart Failure; HD, Heart Disease.

### Procedures

Eligible participants were approached by research staff by phone or onsite to obtain informed consent. As spouses are not directly included in the cardiac rehabilitation program, the consent form and questionnaire package were provided to the patient for their spouse to fill out at home. Written informed consent was obtained from all participants before participating in the study. Research staff were available by phone if potential participants had any questions. Participants were instructed to fill out their questionnaires independently and to return their completed questionnaire packages in person or by mail. The procedures performed in this study were approved by the research ethics board of [masked institution].

### Measures

Participants could choose whether to complete the study questionnaires in English or French. Validated versions of all questionnaires were provided in both languages. In the present sample, nine participants completed the questionnaires in French.

#### Sociodemographic questionnaire

The sociodemographic questionnaire included personal (e.g., age, gender, ethnicity, educational level) and relationship demographics (e.g., marital status, relationship length), as well as clinical information (e.g., physical and mental health conditions, treatment history). Patients’ medical charts were also accessed to confirm cardiac diagnoses and medical history.

#### Experiences in close relationships-12 (ECR-12)

The ECR-12 (Favez et al., 2016 [French]; Lafontaine et al., 2016 [English]) is a 12-item self-report questionnaire that measures two dimensions of adult romantic attachment: anxiety over abandonment and avoidance of intimacy. Example items include: “I worry a fair amount about losing my partner” and “I feel comfortable sharing my private thoughts and feelings with my partner.” Items were rated on a 7-point Likert scale ranging from 1 (*strongly disagree*) to 7 (*strongly agree*). Scores on the anxiety and avoidance subscales were summed. Elevated scores are indicative of higher attachment anxiety or avoidance. Subscale reliability for the present study found comparable indices to the ECR-12 with alpha coefficients of 0.82 and 0.84 for the avoidance and anxiety subscales, respectively.

#### Common dyadic coping (CDC)

Common dyadic coping was assessed with the CDC subscale of the Dyadic Coping Inventory (Bodenmann, 2008 [English]; Ledermann et al., 2010 [French]), which provides information on each partner’s individual perceptions of dyadic coping in their relationship. Example items include: “We try to cope with the problem together and search for ascertained solutions” and “We help one another to put the problem in perspective and see it in a new light.” The five items were rated on a 5-point Likert scale ranging from 1 (*very rarely*) to 5 (*very often*) and summed together to yield a total score, whereby elevated scores are indicative of greater common dyadic coping. Subscale reliability for the present study found an index with a Cronbach alpha coefficient of 0.85, which is comparable to past research (Ledermann et al., 2010; Levesque et al., 2014; Randall et al., 2016).

#### Patient health questionnaire-9 (PHQ-9)

The PHQ-9 (Carballeira et al., 2007 [French]; Kroenke et al., 2001 [English]) is a 9-item scale measuring the presence of nine symptoms of depression within the last 2 weeks. Items were rated on a 4-point Likert scale ranging from 0 (*not at all*) to 3 *(nearly every day)*. Example symptoms include: “little interest or pleasure in doing things,” “feeling tired or having little energy,” and “trouble concentrating on things, such as reading the newspaper or watching television.” The nine items were summed together to yield a total score ranging from 0 to 27, with cut-off points of 5, 10, 15, and 20 representing mild, moderate, moderately severe, and severe levels of depression severity, respectively. The PHQ-9 is a reliable and valid measure of depression among general (Löwe et al., 2008), psychiatric (Beard et al., 2016), and cardiac populations (Beach et al., 2013). Scale reliability for the present study yielded a Cronbach alpha coefficient of 0.81.

#### Generalized anxiety disorder scale-7 (GAD-7)

The GAD-7 (Micoulaud-Franchi et al., 2016 [French]; Spitzer et al., 2006 [English]) is a 7-item self-report measure of generalized anxiety symptoms. Participants rated statements regarding the presence of seven anxiety symptoms within the last 2 weeks. Items were rated on a 4-point Likert scale ranging from 0 *(not at all)* to 3 *(nearly every day)*. The seven items were summed together to yield a total score ranging from 0 to 21, with cut-off points of 5, 10, and 15 representing mild, moderate, and severe levels of anxiety severity, respectively. The GAD-7 is a reliable and valid measure of anxiety among general (Löwe et al., 2008), primary care (Ruiz et al., 2011) and cardiac populations (Conway et al., 2016). Scale reliability for the present study found a Cronbach alpha coefficient of 0.90.

### Data analysis

For the principal analyses, we conducted an actor-partner interdependence mediation model (APIMeM) (Ledermann et al., 2011) in Mplus Version 8.5 (Muthén and Muthén, 2020). We used a structural equation modeling (SEM) framework because it facilitated the estimation of the entire model in one single analysis (Ledermann and Kenny, 2017). APIMeM enables the assessment of mediation in dyadic models while testing the fit of more parsimonious models by constraining actor and partner effects (Peugh et al., 2013). The 6-step procedure developed by Kenny and Ledermann (2010) for APIMeM analysis with distinguishable dyad members was implemented: (1) estimating the saturated distinguishable model and testing of all effects; (2) testing the indistinguishability assumption and specification of a simpler model with direct effects that constrain to equality; (3) estimating the *k’s* and their confidence intervals (Cis) using phantom variables; (4) setting the corresponding *k’s* equal in the event that both the *a* and *b* effects are statistically equal; (5) fixing the *k’s* to 1, 0, or −1 once the CIs values obtained and evaluate the relative fit of the model; and (6) re-specifying the simpler model by constraining effects and eliminating the *k* paths if possible.

In estimating the Actor–Partner Interdependence Model (APIM) (Karney et al., 2005), we fixed the ratio of partner to actor effects (the *k* parameter) at 0.5 for both patient and spouse models. More specifically, the effect of spouse attachment avoidance on patient common dyadic coping was constrained to be half the size of the effect of patient attachment avoidance, and the effect of patient attachment avoidance on spouse common dyadic coping was constrained to be half the size of the effect of spouse attachment avoidance. This specification was guided by prior evidence that actor effects are typically stronger than partner effects in dyadic coping and attachment processes (Falconier et al., 2015; Randall and Bodenmann, 2009), and it allowed us to reduce model complexity and improve estimation stability while still accounting for partner influence (Ledermann and Kenny, 2017).

The fit of the model was determined using several indices. Given the complexity of the present model, the Satorra-Bentler scaled *x*^2^ difference test (2001) was applied, in addition to the Root Mean Square Error of Approximation (RMSEA), the Tucker-Lewis index (TLI), the Comparative Fit Index (CFI), and the Standardised Root Mean Square Residual (SRMSR). According to statistical guidelines, acceptable fit indices are <0.06 for RMSEA, >0.95 for TLI, >0.95 for CFI, and <0.08 for SRMSR (Hooper et al., 2008; Hu and Bentler, 1999). Lastly, differences in the CFI of 0.002 and the Satorra-Bentler scaled *x*^2^ difference test were used to compare nested models (Meade et al., 2008).

We were interested in capturing overall mental health, recognizing that depression and anxiety tend to co-occur and are the most prevalent forms of psychological distress in clinical populations (Kalin, 2020). Given our relatively small sample size, and to reduce the number of estimated parameters, we combined the GAD-7 and PHQ-9 into a composite score. Specifically, we conducted a bifactor analysis with cross-loadings and created a latent variable representing general mental health within the actor–partner model.

## Results

### Descriptive statistics and correlations

**Figure 3:**
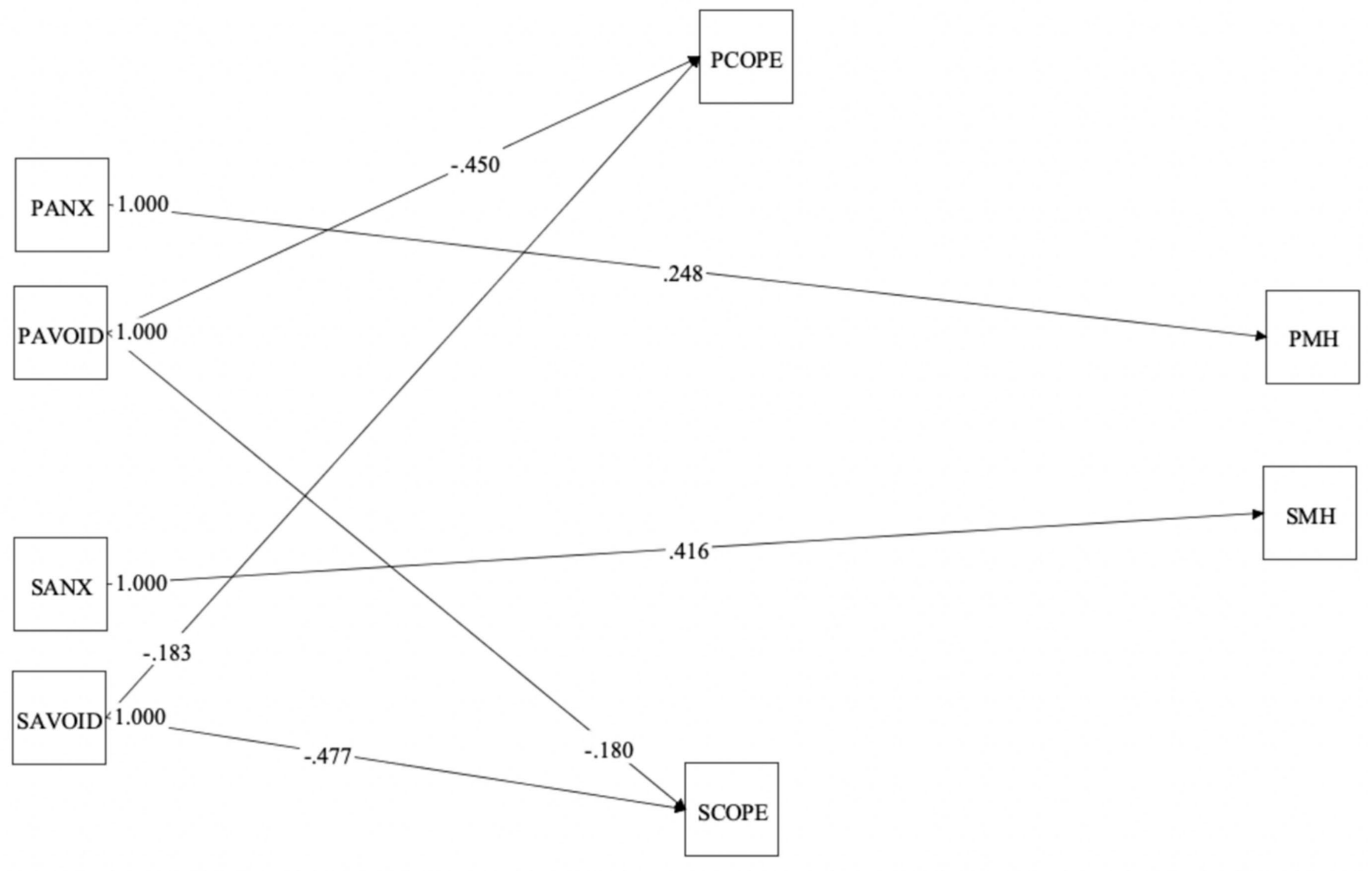
Actor-partner interdependence mediation model relating insecure romantic attachment, common dyadic coping and mental health (i.e., depression and anxiety). PANX, patient attachment anxiety; PAVOID, patient attachment avoidance; SANX, spouse attachment anxiety; SAVOID, patient attachment avoidance; PCOPE, patient common dyadic coping; SCOPE, spouse common dyadic coping; PMH, patient mental health; SMH, spouse mental health. PANX and SANX represent the total attachment anxiety score from the ECR-12 for patients and spouses respectively, whereas PAVOID and SAVOID represent the total attachment avoidance score from the ECR-12 for patients and spouses, respectively.

Overall, less than 5% of data were missing. Little’s missing completely at random (MCAR) test was nonsignificant, *χ*2 (11385) = 11367.124, *p* = 0.545. Means, standard deviations, and Pearson correlations for all studied variables are presented in Table 2 and were in the expected directions. Results of the configural, metric, scalar, and residual invariance for all studied variables showed that most constructs were invariant across members, with the exception of the latent mean invariance for depression and anxiety, which was not supported (*p* = 0.024). This means that the latent means for this variable across groups must be interpreted with caution. The resulting model is displayed in Figure 3, while the actor, partner, direct, and indirect effects are summarized in Table 3.^1^ All significant outcomes are highlighted in Figure 3.

**Table 2:** Pearson correlations for the study variables.

	1	2	3	4	5	6	7	8	9	10
1. Patient attachment avoidance	-									
2. Patient attachment anxiety	0.244**	-								
3. Patient CDC	−0.436**	−0.226**	-							
4. Patient depression	0.246**	−0.106	0.134	-						
5. Patient anxiety	−0.144	−0.135	0.198**	0.601**	-					
6. Spouse attachment avoidance	0.245**	0.08	−0.275**	−0.028	−0.052	-				
7. Spouse attachment anxiety	0.184*	0.298**	−0.138	−0.004	−0.074	0.211**	-			
8. Spouse CDC	−0.287**	−0.132	0.552**	0.121	0.132	−0.443**	−0.156*	-		
9. Spouse depression	−0.003	−0.029	0.039	0.051	0.007	0.03	−0.054	0.01	-	
10. Spouse anxiety	−0.098	−0.032	0.094	−0.035	−0.004	0.009	−0.021	0.034	0.636**	-
M	3.24	2.48	16.93	4.25	3.10	3.39	2.72	16.89	4.43	4.16
SD	0.846	1.258	4.305	3.940	3.427	0.904	1.373	4.244	4.095	4.659

*p < 0.05. **p < 0.01. CDC, Common Dyadic Coping.

**Table 3:** Standardized estimates, bootstrap confidence intervals, and proportion of the total effects.

Effects	Estimate	95% confidence interval	Proportion of total effect (%)
Patient attachment avoidance → patient mental health			
Total effect	0.178	[0.035, 0.311]	100
PAVOID → PCOPE → PMH	0.067	[−0.017, 0.160]	31.02
PAVOID → SCOPE → PMH	−0.014	[−0.067, 0.010]	6.48
Direct effect	0.125	[−0.030, 0.277]	62.50
Patient attachment anxiety → patient mental health			
Total effect	0.267	[0.098, 0.418]	100
PANX → PCOPE → PMH	0.019	[−0.003, 0.070]	7.11
PANX → SCOPE → PMH	−0.001	[−0.026, 0.009]	0.37
Direct effect	0.248	[0.081, 0.406]	92.52
Spouse attachment avoidance → spouse mental health			
Total effect	0.033	[−0.011, 0.283]	100
SAVOID → PCOPE → SMH	0.031	[0.001, 0.087]	39.24
SAVOID → SCOPE → SMH	0.048	[−0.041, 0.153]	60.76
Direct effect	0.054	[−0.112, 0.223]	0 (approx.)
Spouse attachment anxiety → spouse mental health			
Total effect	0.436	[0.276, 0.574]	100
SANX → PCOPE → SMH	0.006	[−0.011, 0.048]	1.38
SANX → SCOPE → SMH	0.014	[−0.006, 0.070]	3.21
Direct effect	0.416	[0.255, 0.566]	95.41
Patient attachment anxiety → spouse mental health			
Total effect	−0.025	[−0.174, 0.116]	100
PANX → PCOPE → SMH	0.022	[−0.001, 0.074]	44.90
PANX → SCOPE → SMH	0.001	[−0.012, 0.032]	2.04
Direct effect	−0.049	[−0.193, 0.095]	53.06
Patient attachment avoidance → spouse mental health			
Total effect	0.013	[−0.143, 0.175]	100
PAVOID → PCOPE → SMH	0.077	[0.000, 0.174]	81.91
PAVOID → SCOPE → SMH	0.018	[−0.011, 0.072]	18.09
Direct effect	−0.082	[−0.245, 0.095]	0 (approx.)
Spouse attachment avoidance → patient mental health			
Total effect	0.019	[−0.130, 0.150]	100
SAVOID → PCOPE → PMH	0.027	[−0.003, 0.085]	41.54
SAVOID → SCOPE → PMH	−0.038	[−0.128, 0.040]	58.46
Direct effect	0.030	[−0.137, 0.177]	0 (approx.)
Spouse attachment anxiety → patient mental health			
Total effect	0.087	[−0.070, 0.238]	100
SANX → PCOPE → PMH	0.006	[−0.010, 0.043]	21.43
SANX → SCOPE → PMH	−0.011	[−0.057, 0.006]	39.29
Direct effect	0.092	[−0.067, 0.247]	39.28

PANX, patient attachment anxiety; PAVOID, patient attachment avoidance; SANX, spouse attachment anxiety; SAVOID, patient attachment avoidance; PCOPE, patient common dyadic coping; SCOPE, spouse common dyadic coping; PMH, patient mental health; SMG, spouse mental health. “0 (approx.)” indicates that the direct effect was very small or nearly zero after proportional distribution.

### Primary analyses

All tested APIM models demonstrated excellent fit to the data. The basic and saturated models yielded perfect fit indices (*χ*^2^ = 0, SRMSR = 0.000, RMSEA = 0.000, TLI = 1.00, CFI = 1.00), as expected. Constrained models that fixed selected k parameters (e.g., k1, k2, k3, k4, k5, k6) also showed very good fit, with *χ*^2^ values ranging from 0.418 to 5.923, SRMSR values ≤ 0.025, RMSEA = 0.000, and TLI/CFI consistently = 1.00. Notably, the more parsimonious models with constraints on k4 (e.g., k4 = −1.5 or −2) provided nearly perfect fit (*χ*^2^ < 1.0, SRMSR < 0.010), indicating that these simplified specifications reproduce the data as well as the fully saturated models while offering a more interpretable structure.

#### Direct effects

a. Effects (*X → M*). These effects pertain to whether a person’s romantic attachment (i.e., how secure or avoidant they are in their relationship) influences how they and their partner handle stress together (common dyadic coping). In terms of patient actor effects, patients’ attachment avoidance (*β* = −0.45, *p* < 0.001) was significantly related to their own common dyadic coping with a medium effect size. The more avoidant they were, the less they tended to engage in common dyadic coping. Similarly for spouse actor effects, spouses’ attachment avoidance (*β* = −0.48, *p* < 0.001) was significantly linked to their own common dyadic coping with a medium effect size. The more avoidant they were, they less they engaged in common dyadic coping. Contrary to our hypotheses, other links were not statistically significant.

Regarding patient partner effects, spouse attachment avoidance (*β* = −0.18, *p = 0.*003) was significantly associated with patient common dyadic coping with a small effect size. Thus, the more avoidant spouses were, the less likely their patient partners were to engage in common dyadic coping. Analogously for spouse partner effects, spouse attachment avoidance (*β* = −0.18, *p = 0.*02) significantly was significantly associated with their own common dyadic coping with a small effect size: The more avoidant spouses were, they less likely they were to engage in dyadic coping themselves. Contrary to our hypotheses, neither patients’ nor spouses’ attachment anxiety were significantly related to their partner’s common dyadic coping.

b. Effects (M → Y). These effects test whether coping together as a couple is linked to the person’s mental health. Contrary to our hypotheses, common dyadic coping was not significantly associated with mental health in any of the four b effects.
c. Effects (X → Y). These effects check whether there is a direct link between a person’s romantic attachment and their mental health, without common dyadic coping as a mediator. Patients’ and spouses’ attachment anxiety were significantly related to their own mental health. As hypothesized, greater patient romantic attachment anxiety (*β* = 0.25, p = 0.003) was related to more of their own symptoms of depression and anxiety with a small effect size, and greater spouse romantic attachment anxiety (*β* = 0.42, *p* < 0.001) was related to more of their own symptoms of depression and anxiety with a medium effect size. Contrary to our hypotheses, neither patients’ nor spouses’ insecure romantic attachment was significantly associated with their partner’s mental health.

#### Indirect effects

Contrary to our hypotheses, common dyadic coping did not mediate the relationship between insecure attachment and mental health in either patients or spouses.

#### Dyadic patterns

Dyadic patterns (e.g., actor-only, partner-only, couple-oriented, and contrast pattern; see Table 3) were estimated by way of *k* parameters. Ten different models were computed in an effort to identify the closest and best fitting values for dyadic patterns. Goodness of absolute fit was found within the last model tested. Results indicated that patient and spouse insecure romantic attachment accounted for 37.1% of the variance in patient common dyadic coping (*R^2^* = 0.371), and 37.5% of the variance in spouse common dyadic coping (*R^2^* = 0.375). Patient and spouse insecure romantic attachment accounted for 18.1% of the variance in patient mental health (*R^2^* = 0.181), and 24.9% of the variance in spouse mental health (*R^2^* = 0.249).

## Discussion

The objectives of the present study were (1) to examine the relationship between insecure romantic attachment and mental health among patients with cardiovascular disease and their spouses, and (2) to explore the potential mediating effect of common dyadic coping. We hypothesized that their own romantic attachment insecurity would be related to lower mental health outcomes in cardiac patients and their partners (H1), that dyadic coping would mediate the path from one’s own romantic attachment security to one’s own mental health (H2), that own insecure romantic attachment would be associated with partners’ lower mental health outcomes (H3), and that dyadic coping would mediate the link from one’s own romantic attachment insecurity to partners’ mental health (H4). The first two hypotheses represent actor effects. The second two represent partner effects.

Our results provide partial support for H1, H2, and H4. As predicted in H1, we found that greater romantic attachment anxiety was related to more symptoms of depression and anxiety in both patients and partners. Specifically, participants’ (patients and partners) own romantic attachment anxiety predicted their own self-reported struggles with their mental health. Contrary to our predictions, we did not detect any effects for attachment avoidance. Concerning H2, we noted that patients’ and spouses’ own romantic attachment avoidance was associated with their own dyadic coping, such that greater avoidance was linked to less CDC. However, we did not detect any relation between attachment anxiety and dyadic coping nor did we uncover any mediating effect of dyadic coping. H3 was not supported: There was no link between individuals’ (patients and spouses) insecure romantic attachment and their partners’ mental health. Finally, there was some support for H4: Spouses’ higher attachment avoidance was related to patients’ lower dyadic coping, but spouses’ attachment anxiety was not related to patients’ dyadic coping. There was also no link between dyadic coping and mental health in any of the analyses.

### Attachment and mental health

Overall, our findings provide further evidence that insecure romantic attachment, specifically attachment anxiety, is linked to one’s own mental health. These results align with longstanding empirical evidence of the link between attachment anxiety and mental health problems in the general population (Mikulincer and Shaver, 2016; Zhang et al., 2022), and with the more recent finding that attachment anxiety in patients participating in cardiac rehabilitation predicts post-traumatic stress, which in turn is related to their symptoms of depression and anxiety both at an initial measurement point and after 3 months (Heenan et al., 2020). It is likely that the context of heart disease, which is often a sudden, life-threatening stressor (Tulloch et al., 2015), activates attachment needs for comfort in both patients and partners, who then either engage their interpersonal supports effectively (secure attachment), become overwhelmed by negative emotion (anxious attachment), or attempt to numb their longings for closeness and connection (avoidant attachment).

The association between anxious attachment and mental health problems that we detected may therefore be attributable to the fact that the chronic reliance on hyperactivating strategies typical of an anxious attachment orientation often impairs the ability of anxiously attached individuals to cope with stressors and to effectively regulate associated negative emotions (Zhang et al., 2022). This would serve to heighten their psychological distress, and it would explain some of their behaviour in the context of heart disease. Consider that in our experience working with couples facing heart disease (Tulloch et al., 2020; Tulloch et al., 2017; Tulloch et al., 2021), patients and partners have mentioned several significant challenges post-diagnosis. In addition to positive relationship changes that in some cases can include greater empathy and a deeper bond, couples also report experiencing sudden role changes (e.g., who handles household chores, finances, caregiving); a need to adapt to new routines that involve diet, exercise, medications, and medical appointments; emotional disconnection and breakdowns in communication; and frequent overprotection of the patient by the partner without heart disease (Tulloch et al., 2020). The dominant emotion when people are faced with the prospect of losing their life partner to heart disease, or when they loathe the possibility of becoming a burden and no longer having a sense of purpose or usefulness in their couple relationship or in their lives, or when they are confronted with their own mortality due to heart disease, is fear (Clarke et al., 2024). Fear is also the dominant emotion in the context of insecure romantic attachment (Domic-Siede et al., 2024). When patients and partners are afraid in this way they pursue, nag, justify, and even withdraw from each other (Tulloch et al., 2017). These behaviors may expose them to more intense symptoms of depression and anxiety due to the deleterious effects they can have on their couple relationship (Landolt et al., 2023).

Attachment avoidance, however, was not significantly linked to mental health in patients or spouses in this study. This was somewhat surprising because attachment avoidance has historically been linked, as has attachment anxiety, to problems with mental health (Mikulincer and Shaver, 2016; Zhang et al., 2022). However, our results do align with recent findings that the association between depressive and anxiety symptoms has been consistently stronger for attachment anxiety than for attachment avoidance (Rapoza et al., 2016; Zheng et al., 2020). The literature highlights that people with an avoidant attachment orientation tend to minimize their problems (e.g., Rapoza et al., 2016; Riggs and Kaminski, 2010). Plus, it is well established that avoidantly attached individuals are likely to use deactivating strategies that inhibit and suppress negative mood from their awareness as a means to cope with negative emotions (Mikulincer and Shaver, 2016). In our study, this tendency may have predisposed patients and spouses with high attachment avoidance to underreport symptoms of depression and anxiety.

Another possibility is that in the short-term aftermath of a cardiac diagnosis (patients in our sample received their diagnosis in the 6 months prior to participating in the study), avoidant strategies might appear to “work” because patients and partners who use them may temporarily numb their distress in order to surmount major challenges. It would be interesting to monitor the long-term effects of these avoidant strategies in patients and partners, because avoidant attachment is known to coincide with dissatisfaction in couple relationships (Li and Chan, 2012), which can increase symptoms of depression (Yang et al., 2023) and anxiety (Postler et al., 2022). Moving forward, it will be important for patients and partners to be aware of the potential impact of their romantic attachment orientations on their mental health. Clinical interventions would therefore do well to attend to attachment anxiety in patients with cardiovascular disease, as in the *Healing Hearts Together* program developed for patients with heart disease and their spouses or life partners (Tulloch et al., 2021).

We were somewhat surprised to find that individual participants’ insecure romantic attachment was not related to partners’ mental health outcomes in patients or spouses. However, there is some evidence in the literature that the actor effects of insecure attachment tend to be stronger than partner effects with regard to people’s mental health. For example, Candel and Turliuc (2019) found in their meta-analysis that insecure attachment was more strongly associated with individuals’ own relationship satisfaction (actor effects) than with their partners’ satisfaction (partner effects). This pattern suggests that attachment insecurity primarily exerts its influence intrapersonally: Individuals’ internal working models and regulatory strategies seem to shape their perceptions of relational security, responsiveness, and fulfillment. Thus, the hyperactivating strategies commonly associated with attachment anxiety or deactivating strategies characteristic of attachment avoidance might heighten personal vulnerability to dissatisfaction through heightened threat sensitivity, negative attribution patterns, or emotional disengagement. The effect of one’s own attachment insecurity on one’s partner was not absent in Candel and Turliuc’s (2019) meta-analysis, however, which suggests that our null finding might also be the result of insufficient power in our sample to detect a similar one.

### Attachment and common dyadic coping

Attachment avoidance was related to dyadic coping in this study. Participants, both cardiac patients and their spouses, who were higher in avoidance tended to engage in less common dyadic coping than did patients who were lower in avoidance. Similarly, patients whose partners demonstrated higher avoidance tended to engage in less common dyadic coping than did patients whose partners were lower in avoidance. Indeed, individuals with high levels of attachment avoidance are less likely to engage in joint coping efforts due to fears of emotional dependency and discomfort with interpersonal closeness, as has been found in a study of members of the general population without a diagnosis of heart disease (Bröning and Wartberg, 2024). It is also likely that patients whose partners tend to avoid engaging them on an emotional level would refrain from turning toward their partner to help manage their stress. This finding has important clinical implications. It suggests that helping partners come together emotionally might help them develop the capacity to develop more effective dyadic coping skills.

Interestingly, and contrary to our expectations, anxious attachment was not significantly associated with CDC in either patients or spouses. According to attachment theory, anxiously attached individuals tend to exaggerate their inability to cope with stressful life events and exhibit strident demands for their partner’s attention, support, and protection. They become focused on their own internal distress, thereby inhibiting their ability to cope effectively with negative emotions (Mikulincer and Shaver, 2016). Individuals with an anxious attachment orientation also have internal working models that bias them to make more negative attributions about their partners and relationships, which may strengthen their perception that conjoint coping efforts are insufficient or inadequate (Campbell et al., 2005). It is also plausible that the presence of this null finding may be explained by the fact that the CDC subscale has a greater portion of items that measure problem-focused rather than emotion-focused strategies. Knowing that anxiously attached individuals tend to use more emotion-focused than problem-focused strategies to manage negative affect (Campbell et al., 2005), the CDC subscale may inaccurately capture hyperactivating strategies which preclude effective dyadic coping.

### Common dyadic coping as a potential mediator

Contrary to our hypothesized mediation model, common dyadic coping did not help to explain the association between any actor or partner effects for romantic attachment avoidance or anxiety and psychological distress. The paucity of mediating effects of common dyadic coping for attachment avoidance could be partially explained by previous evidence. Specifically, individuals scoring high on attachment avoidance tend to rely on compulsive self-reliance as well as emotional, cognitive, and physical distancing from their romantic partner as a means to cope with negative emotions (Mikulincer and Shaver, 2016). For this reason, patients with heart disease might not be inclined to reach for their spouses or life partners when they experience the stress and strain brought about by heart disease. Similarly, partners with avoidant romantic attachment might tend to distance themselves from their feelings about their loved one’s heart disease rather than to engage them in a mutual coping effort.

But anxious attachment in this study was not significantly associated with common dyadic coping in either patients or spouses either, as we thought it would be. Individuals with an anxious attachment orientation are thought to have internal working models that bias them to make more negative attributions about their partners and their relationships, which may strengthen their perception that conjoint coping efforts are insufficient or inadequate (Campbell et al., 2005). It is also plausible that the presence of the null finding may be explained by the fact that the common dyadic coping subscale has a greater portion of items that measure problem-focused rather than emotion-focused strategies. As anxiously attached individuals tend to use more emotion-focused than problem-focused strategies to manage negative affect (Campbell et al., 2005), the common dyadic coping subscale may have inaccurately captured hyperactivating strategies. However, our findings do reflect those of Mosannenzadeh et al. (2024), who noted that people with greater romantic attachment anxiety did not tend to favor interpersonal over intrapersonal emotion-regulation strategies. It is therefore possible that there might be an overall lack of consistency in the preferences of people with anxious romantic attachment with regard to common dyadic coping.

Lastly, neither patient nor spouse common dyadic coping was significantly related to their own mental health. This finding was contrary to our anticipated predictions and to what was found by Kafescioglu et al. (2010), who observed that positive support significantly predicted mental health in both patients and spouses. It is also somewhat contradictory with the findings of Chen et al. (2021), who noted in their literature review of cancer patients and their spouses that positive dyadic coping strategies, including common dyadic coping, were predicitive of positive psychological outcomes. On the other hand, there is some evidence that common dyadic coping is not always associated with decreases in depression. For example, Gellert et al. (2018) found in their study of dyads in which one partner had early-stage dementia that more reported “own” dyadic coping efforts were associated with more depressive symptoms. They also noted that in caregivers dyadic coping measures were less consistently related to their depressive symptoms once other factors were controlled. Another possibility is that common dyadic coping, a construct that emphasizes symmetry in couples’ coping, may be more directly related to relational outcomes, such as relationship quality (Mittinty et al., 2020), than to individual outcomes, such as depression and anxiety.

## Limitations and future directions

There are several strengths to this study, including the measurement of both patients’ and spouses’ perceptions of their romantic attachment, common dyadic coping, and mental health; the use of validated questionnaires with robust psychometric properties; the addition of common dyadic coping as a potential mediating construct; and the analysis of patient and partner effects using as sophisticated model that accounts for interdependent relationships. Although this research addresses gaps in the literature and has theoretical and methodological strengths, there are limitations that should be noted. First, the cross-sectional nature of this study precludes any observations of causality, although it is still advantageous preliminary work that is necessary before embarking on more resource-intensive experimental research (Czajkowski et al., 2015). Second, despite our best attempts to recruit a diverse sample, the characteristics of the participants in this study were relatively homogenous, including mostly white, well-educated, male patients and female spouses aged 60 and over, with an average relationship length of more than 30 years. It is possible that people in this age group perceive and experience attachment and mental health differently than do people who are younger or older, just as it is possible, even likely, that people of sociocultural or ethnic backgrounds that do not represent the majority might have different experiences with attachment and mental health (Guillory, 2021). Our understanding of the associations between attachment and mental health would benefit greatly from samples that are more diverse in terms of socioeconomic status, race/ethnicity, education, sex, and age, as these factors are all implicated in the association between relationship quality and mental health (Lincoln and Chae, 2010; Yu and Zhang, 2020).

Furthermore, the participant population was recruited from a cardiac rehabilitation program. Participation rates for cardiac rehabilitation at our center are approximately 50% of those diagnosed with cardiovascular disease, and these participants typically have fewer comorbidities than do non-participants (Peters and Keeley, 2018). In this study, only a small subset of participants (13.5%) reported poor mental health, which may partially explain some of the null findings. Low levels of anxiety and depression may also be a result of participation in a cardiac rehabilitation program that included elements of stress management; therefore, it is plausible that participants’ anxiety and depression symptoms would be lower than those who did not participate in cardiac rehabilitation (who were not participants in this study). Finally, although the CDC subscale has been found to have good psychometric properties, it is plausible that its brevity may not have entirely captured how relational coping efforts possibly influence mental health, hence limiting the measured variance. The same holds true for our measures of attachment and mental health: all were brief and might not provide a completely accurate snapshot of the experiences of participants. Future research would do well to include measures from multiple sources (e.g., diagnoses from mental health professionals, observational measures of couple interactions) beyond the self-report measures included here. It would also be good to compare couples facing heart disease with couples in good physical health to determine which aspects of dyadic coping, attachment and mental health might be specific to the context of heart disease.

## Conclusion

The effects in this cross-sectional study, although only small to moderate, indicate that insecure attachment, particularly attachment anxiety, may be linked to symptoms of depression and anxiety following a cardiovascular event or diagnosis. This provides scientific justification for further examination of attachment and mental health in clinical samples using longitudinal designs. Our results additionally revealed that common dyadic coping was not a significant mediator of the relationship between insecure romantic attachment and depression and anxiety. Further research investigating common dyadic coping and other relational factors (e.g., relationship satisfaction, intimacy, mutual disclosure) is now warranted. Relationship quality and coping skills are modifiable factors that are sensitive to intervention (Berry et al., 2017). They therefore represent an excellent opportunity for secondary prevention.

Although no partner effects for our main objective were observed in this study, decades of research have illuminated the potential “spill over” effects of an individual’s health on family members and shown that these effects are reciprocal (Wittenberg and Prosser, 2016). Thus, there is still a continued need for research to assess and analyze patient and spousal effects interdependently. In a cardiovascular disease context, this shift from individual-focused to a dyadic- or family-focused health management has led to increased understanding of the ways that spouses’ behaviors and outcomes influence those of patients and vice-versa (Bidwell et al., 2023). Accumulating research also seems to indicate that couples-or family-based interventions (that target both the patient and a loved one) yield sustainable effects on mental health, quality of life, and cardiometabolic risk factors (Helgeson et al., 2018). To further inform such interventions, future research, particularly longitudinal studies, should aim to clarify the processes and potential mechanisms that explain the interconnections between couples’ physical and mental health, in which relational factors appear to figure prominently.

## Data Availability

The raw data supporting the conclusions of this article will be made available by the authors, without undue reservation.

## Footnotes

1 Note: The generative artificial intelligence software ChatGPT (OpenAI, 2023) was used to edit Table 3.
